# Supplementary material for: Comprehensive assembly of novel transcripts from unmapped human RNA-Seq data and their association with cancer
Source: Mol Syst Biol. 2015 Aug 7;11(8):826. doi: 10.15252/msb.156172 (PMC4562499; doi:10.15252/msb.156172)
Supplement: Supplementary file 1 — Appendix [file msb0011-0826-sd1.pdf]

Appendix

Comprehensive assembly of novel transcripts from unmapped human RNA-Sequencing data and their association with cancer

Majid Kazemian, Min Ren, Jian-Xin Lin, Wei Liao, Rosanne Spolski, Warren J. Leonard

Laboratory of Molecular Immunology and the Immunology Center

National Heart, Lung, and Blood Institute

National Institutes of Health

Bethesda, MD 20892-1674

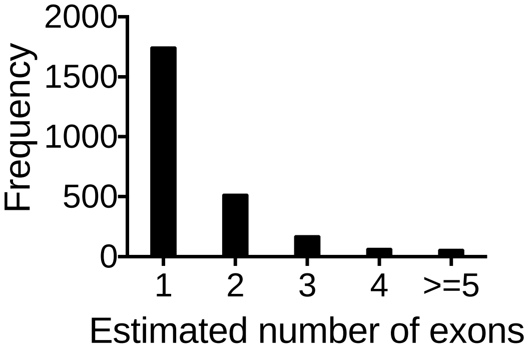

Appendix Figure S1. Distribution of the number of exons in 2550 long human transcripts. Transcripts were aligned against human, chimp, and gorilla genomes using BLAT (see **Table EV4**), and the number of gaps was determined. The number of exons was set as the number of gaps + 1 from the target genome with the best BLAT score, as gaps represent potential introns. However, it is possible that some of the gaps in chimp and gorilla do not correspond to the human introns; thus, the number of exons is an estimate pending precise assembly of these transcripts in the human genome.

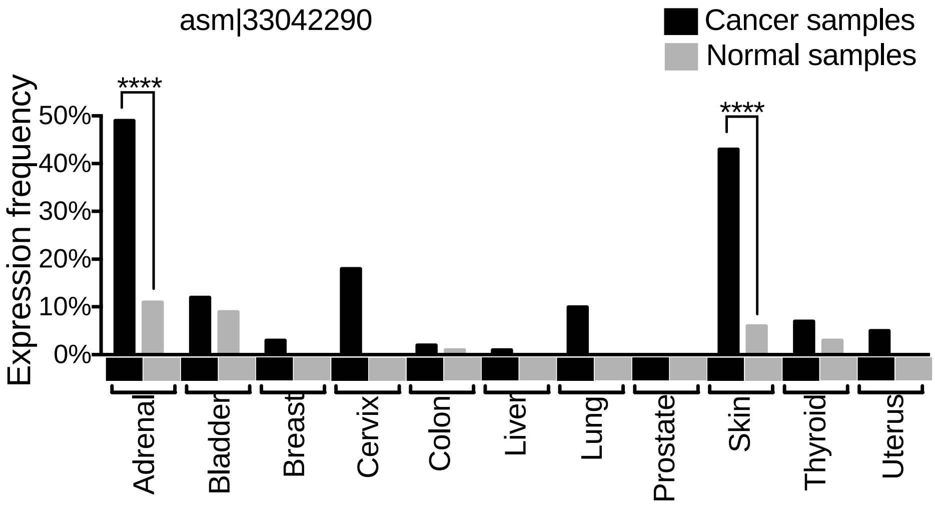

Appendix Figure S2. Expression frequency of asm|33042290 in various cancer and normal tissue samples. Significance was calculated using Fisher's exact test on a two-by-two contingency table, representing the number of normal or cancer samples in a given tissue that express or do not express transcript asm|33042290.

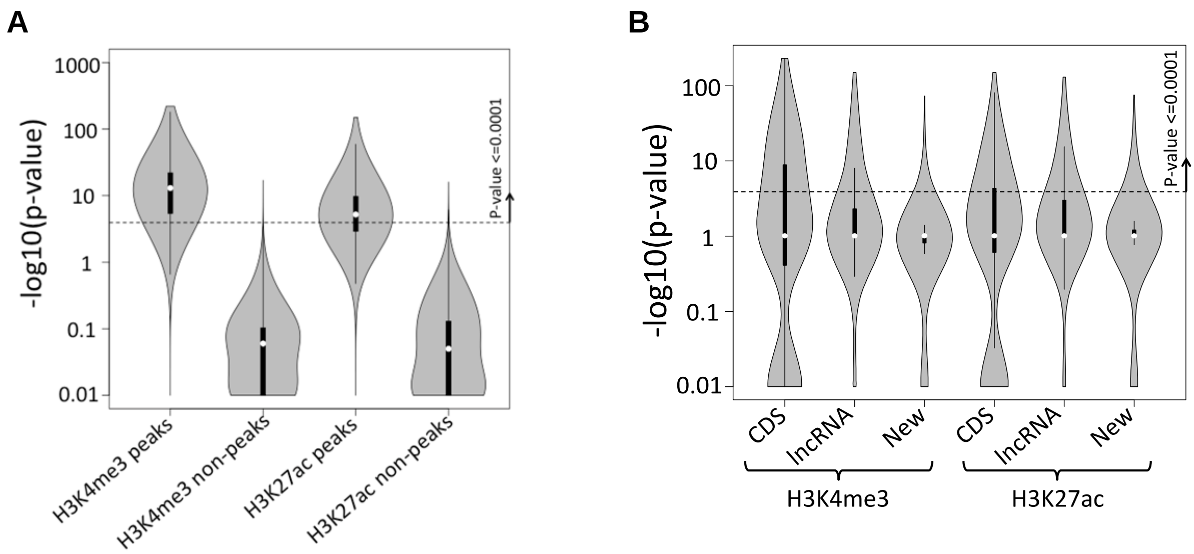

Appendix Figure S3. Calling histone marks.

A Distribution of predicted scores for H3K4me3 and H3K27ac peak and non-peak regions. Shown is the significance of the scores from our method (see the “Calling histone marks at genomic loci of newly assembled transcripts” in Materials and Methods) for all peaks and non-peaks identified by MACS program on histone H3K4me3 and H3K27ac ChIP-Seq data from 11 cancer cell lines used in this study. The p-value cutoff (0.0001) used in this study is marked by the dashed-line, corresponding to 99.98% specificity and >60% sensitivity.

B Violin plot comparing the predicted scores for histone H3K4me3 and H3K27ac across gene categories. CDS corresponds to 17529 protein-coding transcripts. lncRNAs represent 2098 known noncoding RNAs from RefSeq database. “New” corresponds to 2550 transcripts found in this study.
