# Supplementary material for: Comprehensive assembly of novel transcripts from unmapped human RNA-Seq data and their association with cancer
Source: Mol Syst Biol. 2015 Aug 7;11(8):826. doi: 10.15252/msb.156172 (PMC4562499; doi:10.15252/msb.156172)
Supplement: Supplementary file 2 — Expanded View Figures PDF [file msb0011-0826-sd2.pdf]

Expanded View Figures

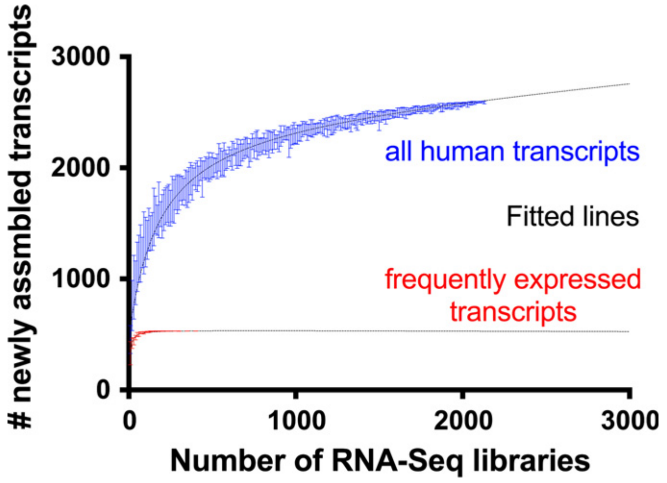

Figure EV1. Simulation of the number of newly assembled transcripts as a function of the number of RNA-Seq samples.
For each number of RNA-Seq libraries analyzed (sample size), the estimated number of newly assembled human transcripts that would be identified via our pipeline is shown. The fitted lines are based on the nonlinear "One site" binding equation. For each sample size, 10 random subsets of normal and cancer patient samples were selected, and the corresponding number of assembled transcripts, represented by error bars, was calculated.

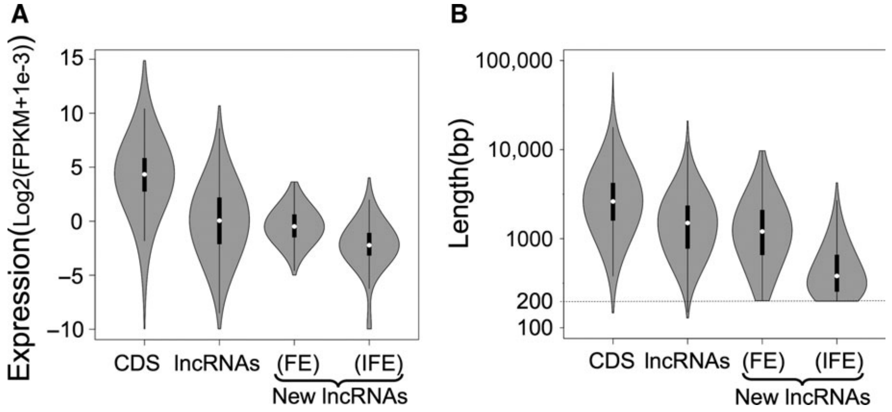

Figure EV2. Comparing the length and expression of newly assembled transcripts with transcripts in other categories.

- A Violin plot comparing the maximum expression of each transcript in all cancer samples across gene categories. CDS corresponds to 17,529 protein-coding transcripts. lncRNAs represent 2,098 known noncoding RNAs from RefSeq database. FE and IFE: 521 frequently and 2,029 infrequently expressed transcripts, respectively, found in this study.
- B Violin plot comparing the length of transcripts across gene categories. 200 bp was the length cutoff used for identifying these transcripts.

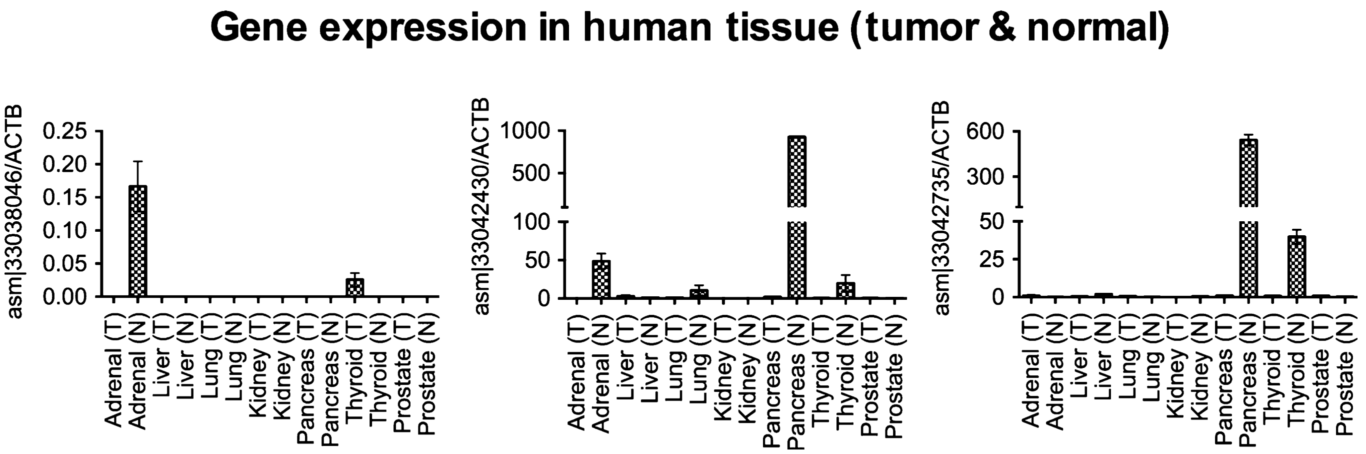

Figure EV3. Expression of transcripts asm|3308046, asm|33042430, and asm|33042735 in various cancer and normal tissue samples.
Shown is the relative expression of three indicated transcripts in 7 cancer (T) and normal (N) tissues. Error bars show standard error of the mean of two replicates.

This article is a U.S. Government work and is in the public domain in the USA

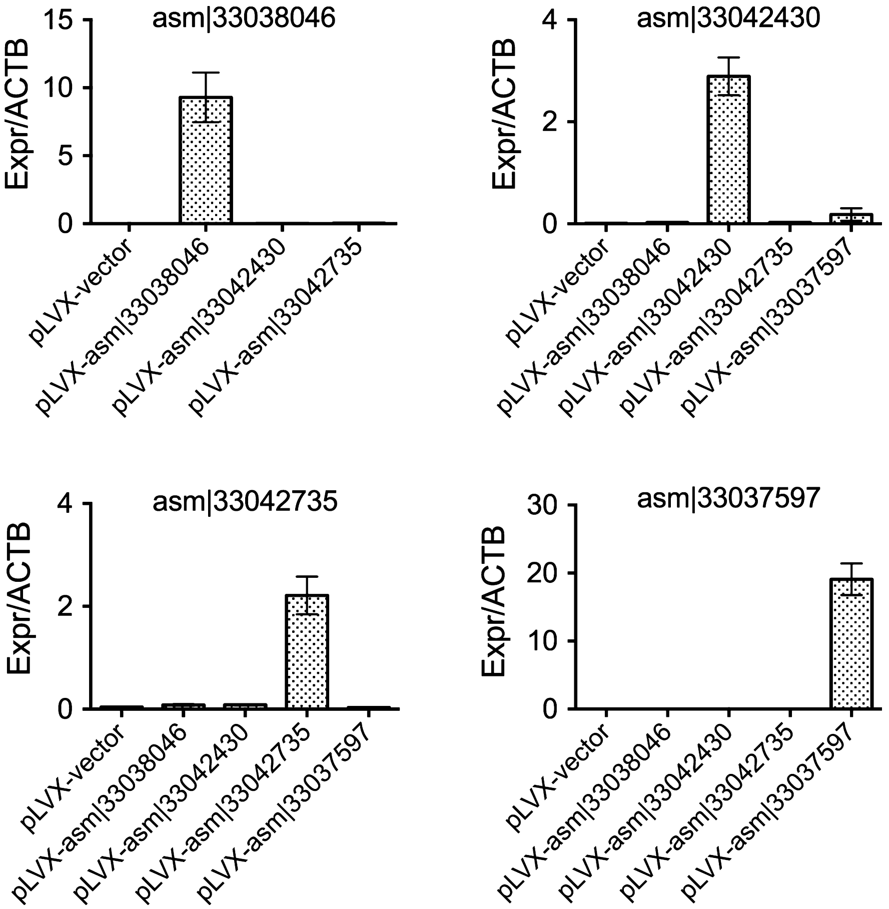

Figure EV4. Overexpression of transcripts in HepG2 cells.
Shown is the relative expression of each of the indicated transcripts to ACTB in HepG2 cells. Error bars show standard error of the mean of two replicates.

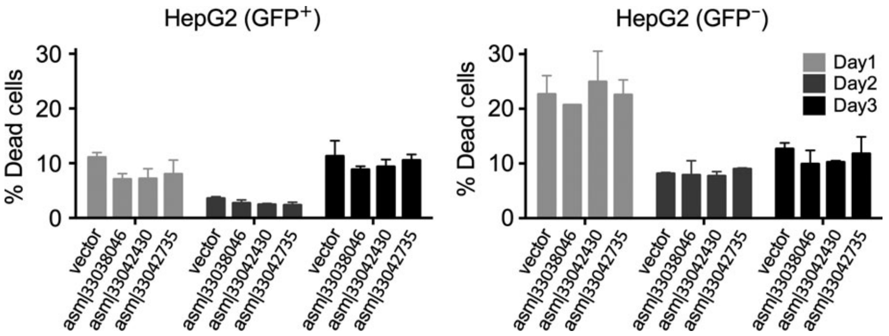

Figure EV5. Cell viability assay.
Shown are the percentages of dead cells in transduced (left) and non-transduced (right) cells, as measured by flow cytometry using viability dye over the course of 3 days. Error bars show standard deviation of the mean of two replicates.

This article is a U.S. Government work and is in the public domain in the USA
