# Supplementary material for: Comprehensive assembly of novel transcripts from unmapped human RNA-Seq data and their association with cancer
Source: Mol Syst Biol. 2015 Aug 7;11(8):826. doi: 10.15252/msb.156172 (PMC4562499; doi:10.15252/msb.156172)
Supplement: Supplementary file 6 — Table EV4 [file msb0011-0826-sd6.docx]

**Table EV4. Nucleotide BLAST of 8 selected transcripts against human and chimp genomes.**

Shown are nucleotide BLAST statistics against homo sapien (GRCh38) and chimp (Pan_tro-2.1.4) genomes. The statistics include BLAST total score, the query coverage (i.e., the percentage of transcripts that were aligned), BLAST E value, and identity (i.e., the percentage of nucleotide identity for the covered region).

|  | **Homo sapiens (GRCh38)** | | | | **Chimp (Pan_tro-2.1.4)** | | | |
| --- | --- | --- | --- | --- | --- | --- | --- | --- |
| **Transcript ID** | **Total score** | **Query coverage** | **E value** | **Identity** | **Total score** | **Query coverage** | **E value** | **Identity** |
| **asm\|33042367** | - | - | - | - | 1480 | 100% | 0 | 99% |
| **asm\|33043058** | 82.4 | 18% | 7E-13 | 73% | 2365 | 91% | 0 | 99% |
| **asm\|33042430** | - | - | - | - | 1348 | 93% | 0 | 99% |
| **asm\|33038046** | 159 | 3% | 7E-13 | 100% | 4510 | 100% | 0 | 99% |
| **asm\|33042196** | - | - | - | - | 1457 | 94% | 0 | 98% |
| **asm\|33037597** | - | - | - | - | 1031 | 100% | 0 | 98% |
| **asm\|33042655** | 76.8 | 1% | 3E-11 | 100% | 4592 | 96% | 0 | 99% |
| **asm\|33042735** | - | - | - | - | 1269 | 93% | 0 | 99% |
