# Supplementary material for: Comprehensive assembly of novel transcripts from unmapped human RNA-Seq data and their association with cancer
Source: Mol Syst Biol. 2015 Aug 7;11(8):826. doi: 10.15252/msb.156172 (PMC4562499; doi:10.15252/msb.156172)
Supplement: Supplementary file 11 — Table EV9 [file msb0011-0826-sd11.docx]

**Table EV9. Association between histone marks and expression.**

A contingency table showing the number of transcripts in four categories: transcripts that are frequently expressed or not (or infrequently) expressed and have or do not have histone marks in their genomic loci from matching tissue. 188 transcripts with at least one significant histone mark in breast, cervix, colon, liver, lung, and prostate cell lines were evaluated. The table summarizes these 6 x 188 = 1128 possibilities.

|  | Histone mark | No histone mark |
| --- | --- | --- |
| Frequently expressed | 65 | 87 |
| Not (or infrequently) expressed | 251 | 725 |
